# A systematic review and meta-analyses of pregnancy and fetal outcomes in women with multiple sclerosis: a contribution from the IMI2 ConcePTION project

**DOI:** 10.1007/s00415-020-09913-1

**Published:** 2020-05-22

**Authors:** Sandra Lopez-Leon, Yvonne Geissbühler, Meritxell Sabidó, Moise Turkson, Charlotte Wahlich, Joan K. Morris

**Affiliations:** 1 Novartis Pharmaceuticals Corporation, One Health Plaza, Building 339-1131, East Hanover, NJ 07936-1080 USA; 2 Novartis Pharma AG, Basel, Switzerland; 3 Global Epidemiology, Merck KGaA, Darmstadt, Germany; 4 Cognizant Solution, London, UK; 5 Population Health Research Institute, St George’s, University of London, London, UK

**Keywords:** Multiple sclerosis, Pregnancy, Congenital malformations, Preterm, Spontaneous abortions

## Abstract

Neurologists managing women with Multiple Sclerosis (MS) need information about the safety of disease modifying drugs (DMDs) during pregnancy. However, this knowledge is limited. The present study aims to summarize previous studies by performing a systematic review and meta-analyses. The terms “multiple sclerosis” combined with DMDs of interest and a broad profile for pregnancy terms were used to search Embase and Medline databases to identify relevant studies published from January 2000 to July 2019.1260 studies were identified and ten studies met our inclusion criteria. Pooled risk ratios (RR) of pregnancy and birth outcomes in pregnancies exposed to DMDs compared to those not exposed were calculated using a random effects model. For spontaneous abortion RR = 1.14, 95% CI 0.99–1.32, for preterm births RR = 0.93, 95% CI 0.72–1.21 and for major congenital malformations RR = 0.86, 95% CI 0.47–1.56. The most common major congenital malformations reported in MS patients exposed to MS drugs were atrial septal defect (ASD) (*N =* 4), polydactyly (*N =* 4) and club foot (*N =* 3), which are among the most prevalent birth defects observed in the general population. In conclusion, interferons, glatiramer acetate or natalizumab, do not appear to increase the risk for spontaneous abortions, pre-term birth or major congenital malformations. There were very few patients included that were exposed to fingolimod, azathioprine and rituximab; therefore, these results cannot be generalized across drugs. Future studies including internal comparators are needed to enable treating physicians and their patients to decide on the best treatment options.

## Introduction

Multiple sclerosis (MS) is a chronic neurological disease that affects more women than men, with a female-to-male ratio of 2:1 [1]. MS is generally diagnosed during childbearing age [1]. Symptoms associated with MS may occur in isolated attacks or build up over time. Between attacks, symptoms may disappear completely; however, permanent neurological problems often remain. Hence, the initiation of early-treatment with disease modifying drugs (DMDs) is recommended [2].

Neurologists managing women with MS who wish to conceive need to use a benefit-risk approach and provide adequate advice to their patients. Physicians also need to be prepared in case women taking DMDs become pregnant given that many pregnancies occur unplanned, and therefore, women with MS may inadvertently take DMDs whilst pregnant. It is essential to balance the risk of DMDs during pregnancy to the fetus as compared to the risk of inadequate treatment in the mother. During pregnancy lack of treatment may lead to the development of irreversible disability, since stopping an efficacious drug may induce a relapse [3].

Despite previous systematic reviews and studies [4–6], knowledge about the consequences of treatment with DMDs during pregnancy is limited. Based on animal studies and as a result of limited data in humans, the Food and Drug Administration (FDA) assigned category C to the use of most of the DMDs during pregnancy. This category states: “Risk not ruled out: Animal reproduction studies have shown an adverse effect on the fetus and there are no adequate and well-controlled studies in humans, but potential benefits may warrant use of the drug in pregnant women despite potential risks”. The use of pregnancy risk categories presented a challenge for many health care providers, since it did not include enough detailed information related to the safety and efficacy of medications in pregnancy and lactation to assist them in making more evidence-based decisions. As a result, in December 2014 FDA introduced the Pregnancy and Lactation Labeling Rule (PLLR) which removed the pregnancy letter categories. In place of these pregnancy categories, the PLLR requires narrative explanations of risk and supporting data [7]. The EMA provides a decision scheme that helps determine whether or not a contraindication during pregnancy should be settled in the labelling [8]. This guideline considers all non-clinical and clinical data to make an integrated approach in the risk assessment. In recent years, the landscape of MS treatment has changed, with an increasing number of treatment options that have a long-lasting disease modifying effect. Due to the longer half-lives, there is potentially a longer impact during pregnancy. Studies in pregnant women with newer DMDs are scarce.

The aim of this meta-analysis is to evaluate pregnancy outcomes in women with MS treated with DMDs. The results will add to the existing evidence for women with MS considering pregnancy and for neurologists deciding on treatment around pregnancy and counselling women with unplanned pregnancies. This study will also help understand what evidence is lacking in order to provide guidance on what to consider when performing future studies that evaluate clerosis MS treatment during pregnancy.

## Methods

### Search strategy

The PRISMA 2009 guidelines were followed throughout the study [9]. Searches in Embase and Medline were conducted May–August 2019 to identify relevant publications from the period January 2000 to August 2019. The term multiple sclerosis was combined together with DMDs of interest with a broad profile for pregnancy terms. DMDs of interest were: interferon β-1a, interferon β-1b, PEG interferon β-1a, alemtuzumab, cladribine, dimethyl fumarate, fingolimod, glatiramer acetate, laquinimod, natalizumab, teriflunomide, methotrexate, cyclophosphamide, mycophenolate mofetil, azathioprine, rituximab, mitoxantrone teriflunomide, laquinimod, natalizumab, ofatumumab, rituximab, ocrelizumab, disease modifying, DMD, DMDs B-cell therapy. The pregnancy terms used were: pregnancy, gravidity, prenatal, maternal, gestation, pregnancies, pregnant, pregnanc*, fetal, foetal and congenital. The search was restricted to publications in English. The search was performed using the databases’ controlled vocabulary as well as free-text terms including various spellings and synonyms. Duplicates were removed.

### Eligibility criteria

Titles and abstracts were screened to identify peer-reviewed studies which measured the effects of MS drugs on pregnancy. Only studies in which the exposure occurred in utero and compared patients exposed to an MS drug against MS patients without treatment or against the general population were included. Studies which mixed unexposed and exposed in the same group were excluded (e.g., MS patients with and without treatment). However, if the studies included patients taking different DMDs and compared them with one control group they were included as a general DMD group. Interventional studies (e.g., randomized clinical trials) as well as non-interventional studies (e.g., cohort, case–control, and registries) were included.

To avoid bias, studies where the pregnancy outcome was known when the patient entered the study (e.g., retrospective cases) were excluded. Therefore, the pregnancy outcomes were assessed independently of the knowledge regarding pregnancy exposure.

Both primary data collection (e.g., registry studies) as well as studies that included secondary sources (e.g., claims databases) of data were considered. Abstract, reviews, case reports, case series and spontaneous reports were excluded. There was no exclusion based on the pregnancy outcomes, all outcomes available were included.

### Data extraction

Two reviewers (MT, MS) independently reviewed the search results for inclusion narrowing potential studies successively in three stages: by title, by abstract, and by full manuscript. Disagreements regarding eligibility were discussed amongst all authors. The quality of eligible studies was independently assessed by SLL and CW using the New Castle Ottawa scale (NOS) [9]. Studies with NOS scores greater than six points were considered to be of high quality. Data from all selected articles were extracted by two authors (MT and SLL) and a quality control, by detecting inconsistencies, was performed by another author (CW).

### Statistical analysis

The primary analyses consisted of calculating pooled risk ratios (RR) of pregnancy and birth outcomes (spontaneous abortions, pre-term birth and congenital malformations) in pregnancies exposed to multiple sclerosis drugs compared to those not exposed. All MS drugs were studied together overall, and when possible stratified by drug class [interferons, natalizumab and glatiramer acetate (GA)] to study each individual DMD separately. The pooled RR of all meta-analyses were calculated using a random effects model. The relative weight for each study is calculated as the inverse of the sum of the individual study variance plus the between study variance. The weight for each study is then the relative weight expressed as a proportion of all the relative weights of the studies in the analysis. Therefore, the weight for any study will vary according not only to the number of events occurring in that study and the size of the study, but also to the number of events and size in other studies and also how consistent the study effect is between the studies. The data were analyzed using STATA Version 15.

The secondary analyses consisted of calculating the pooled prevalence rate of the pregnancy outcomes which had two or more published studies in untreated as well as treated MS patients. MetaXL software was used to estimate the pooled prevalence which uses a double arcsine transformation [10].

For all outcomes, except congenital malformations, “all pregnancies” (live birth, spontaneous abortion, or termination of pregnancy) were used as the denominator. In relation to major congenital malformations, all studies used “live births”; therefore, live births were used as the denominator. Heterogeneity was assessed using the *I*^2^ statistics. Values of 25%, 50% and 75% for *I*^2^ represented low, medium and high heterogeneity, respectively [11]. Publication bias was considered using funnel plots and assessed with Harbord's modified test for small-study effects.

## Results

### Study selection and characteristics

**Fig. 1:**
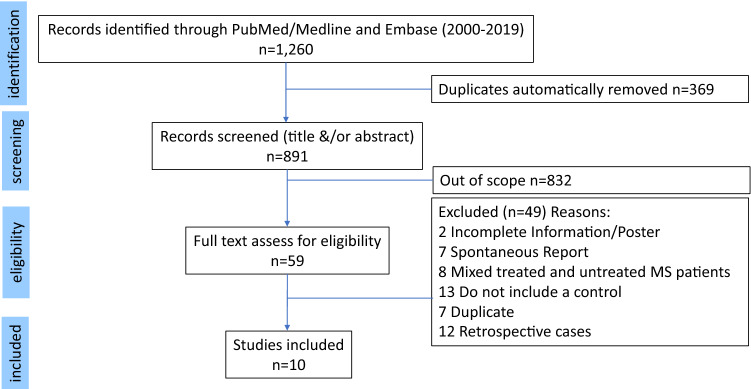
Flowchart depicting the study selection and record screening process

The study search and selection processes are described in detail in Fig. 1. The initial search yielded 1260 publications. After removing duplicates 891 publications were identified. The titles and abstracts were then screened; 832 studies were out of scope and 59 studies were assessed in full. Only ten studies met our inclusion criteria. These ten studies were considered to be of high quality based on the NCO scale (NOS) and were included in the meta-analyses.

### Studies included

Table 1 provides characteristics of the included studies. The studies were from Germany (*N =* 4), Canada (*N =* 2), Spain, Italy, US and Worldwide. The studies from Germany came from the same cohort; however, the data were not duplicated as the studies selected included different dates or medications. All of the studies were observational studies.

**Table 1:** General characteristics

Author (year)	Country, study period	Study design	Population based	Data source: name, type	Source of exposure information	Source of outcome information	N MS pregnant women included
Boskovic et al. [23]	Canada, 1997–2004	Longitudinal cohort	No, spontaneous contact /enrolment	Motherisk program	Teratogen information and counseling service at the Hospital for Sick Children	Reported by mother and verified, by the child’s pediatrician or family physician,	16 IFN 12 Unexposed
De las Heras et al. [11]	Spain, period not reported	Cohort, secondary use of data^a^	No	NA, EMR	Neurological services of 16 hospitals	Same service	34 Immunomodulator 54 Unexposed
Weber-Schoendorfer and Schaefer [24]	Germany, 1996–2007		No, spontaneous contact/enrolment	TIS Berlin	Teratology Information Service	Reported by mother and verified by her physician and/or the pediatrician	31 GA 69 IFN 64 Unexposed
Lu et al. [25]	Canada, 1998–2009	Cohort, secondary use of data through linkage	Yes provincial coverage	British Columbia (BC) MS database BC Perinatal Database Registry EHR	British Columbia MS database	BC Perinatal Database Registry	15 IFN 6 GA 317 Unexposed
Ebrahimi et al. [16]	Germany, 2006–2013	Longitudinal cohort	No, national coverage	German pregnancy MS registry	Either through women responding to advertisements or actively recruited after referral by their treating clinicians or MS nurses	Telephone interview every 3 months or visits to the university-based outpatient clinic in Bochum	101 Natalizumab No proper control for this meta-analysis (mixed treated (other than natalizumab) and untreated MS patients)
Herbstritt et al. [13]	Germany, 2008–2013	Longitudinal cohort	No national coverage	German pregnancy MS registry, registry	Either through women responding to advertisements or actively recruited after referral by their treating clinicians or MS nurses	Telephone interview every 3 months or visits to the university-based outpatient clinic in Bochum	151 GA 95 Unexposed
Thiel et al. (2016) [14]	Germany, January 2008 –December 2013	Longitudinal cohort	No national coverage	German pregnancy MS registry, registry	Either through women responding to advertisements or actively recruited after referral by their treating clinicians or MS nurses	Telephone interview every 3 months or visits to the university-based outpatient clinic in Bochum	251 IFN 194 Unexposed
Portaccio et al. [15]	Italy, 2009–2015 and 2002–2008	Longitudinal cohort	Yes	NA, EMR	Neurological services of 19 hospitals	Collected by neurologist during patients visits	62 Natalizumab 87 Interferon 332 Unexposed
MacDonald [12]	US, 2011–2015	Cohort secondary use of data^a^	Yes national coverage	Truven MarketScan database, claims	Commercially insured population and recorded in the data source	Infants kept in health plan of the mother and recorded in the data source	574 DMD overall^b^ 225 GA, 118 IFN, 39 Natalizumab, 19 DF, 18 Fingolimod 1075 Unexposed
Nguyen et al. [26]	Worldwide, 2005–2016	Cohort, secondary use of data^a^	Yes	MSBase, EHR	Sites participating and entering data into MSBase	Same	1178 DMD overall 350 IFN, 137 GA, 104 Natalizumab, 17 DF, 21 Fingolimod, 4 Azathioprine, 2 Rituximab/B-cell depletion, 886 Untreated

*DMD*  disease modifying drugs, *DF*  dimethyl fumarate, *EHR* electronic health record, *EMR* electronic medical record, *GA* glatiramer acetate, *IFN*  interferon, *MS*  multiple sclerosis, *NA* not applicable

^a^Secondary use of data corresponds to data extraction

^b^Authors did not stratify by type of DMD

The drugs that were studied the most were interferon, glatiramer acetate, natalizumab. There were very few patients included that were treated with fingolimod, azathioprine or rituximab, MacDonald et al. [12] did not stratify their analyses by type of drug and only conducted their analyses as overall. Therefore, it was not possible to include their data in the drug-specific analyses. Five of the studies only included patients exposed to DMD during the first trimester [5, 13–16]. None of the studies stratified by trimester of exposure. All of the studies, except one, included as control patients with multiple sclerosis that were not exposed to disease modifying drugs. The comparison group in the study by Ebrahimi et al. [16] included a mixture of multiple sclerosis patients treated with other DMDs than natalizumab and untreated MS patients. Therefore, the data from this study were only included in the pooled estimated prevalence but not in the meta-analysis of risk ratios.

The birth outcomes in which there were enough studies to conduct a meta-analysis were spontaneous abortions, pre-term births and congenital malformations. All studies defined spontaneous abortions as fetal loss before week 22nd of pregnancy. Pre-term birth was defined in all studies as birth before week 37th of pregnancy. All of the studies did not give a definition for adjudication of the malformations nor stated what system they used to define them, they just referred to them as “major malformations”, “major structural malformations” or “major birth defects”. There was no standardization in the definition or wording used. All of the studies, except one, specified that they were major malformations. The study that only referred to them as “congenital malformations” reported that there were none present [13].

### Meta-analysis of risk ratios

**Fig. 2:**
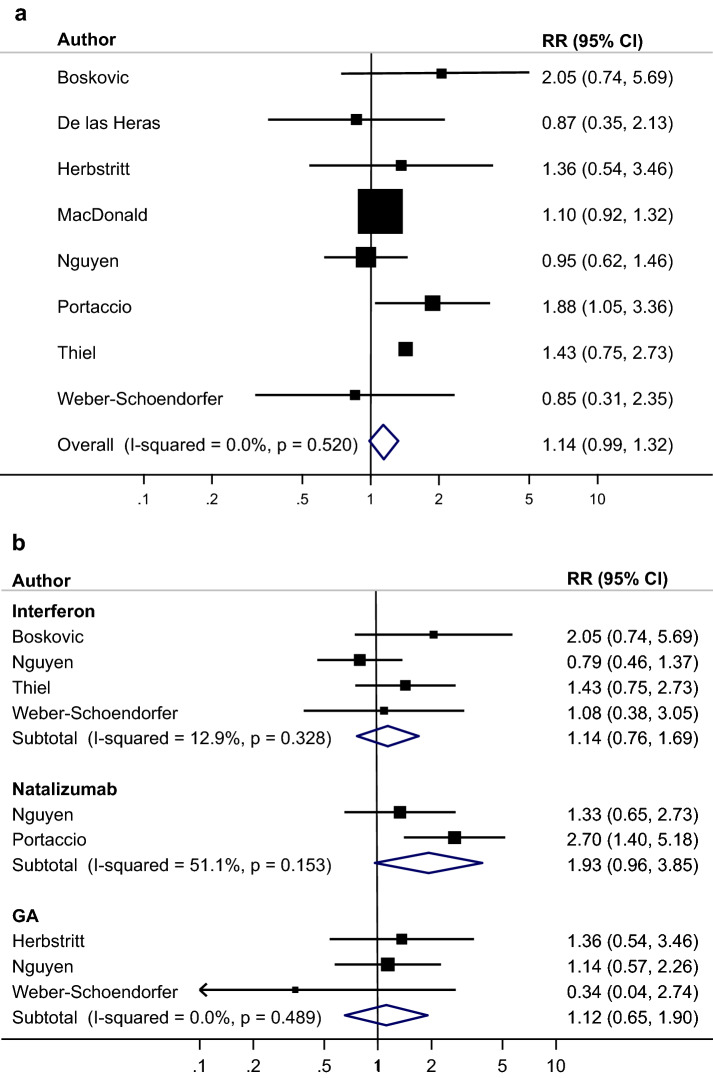
**a** Meta-analysis spontaneous abortions: treated MS vs untreated MS. **b** Meta-analysis spontaneous abortions: treated MS vs untreated MS stratified by drug

**Fig. 3:**
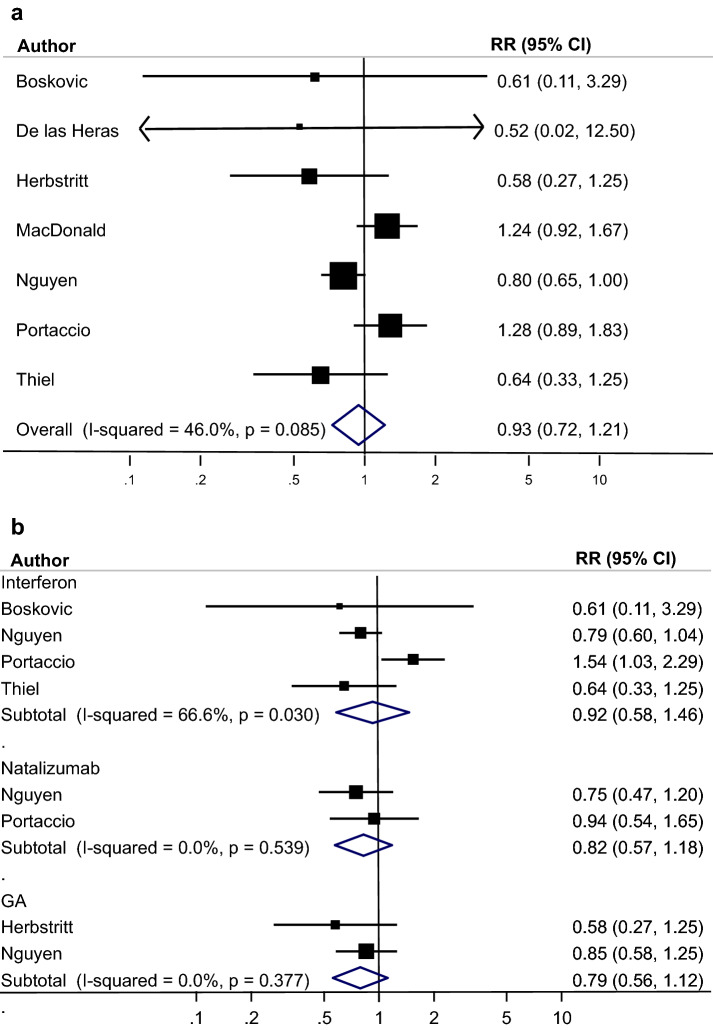
**a** Meta-analysis pre-term birth: treated MS vs untreated MS. **b** Meta-analysis pre-term birth: treated MS vs untreated MS stratified by drug

**Fig. 4:**
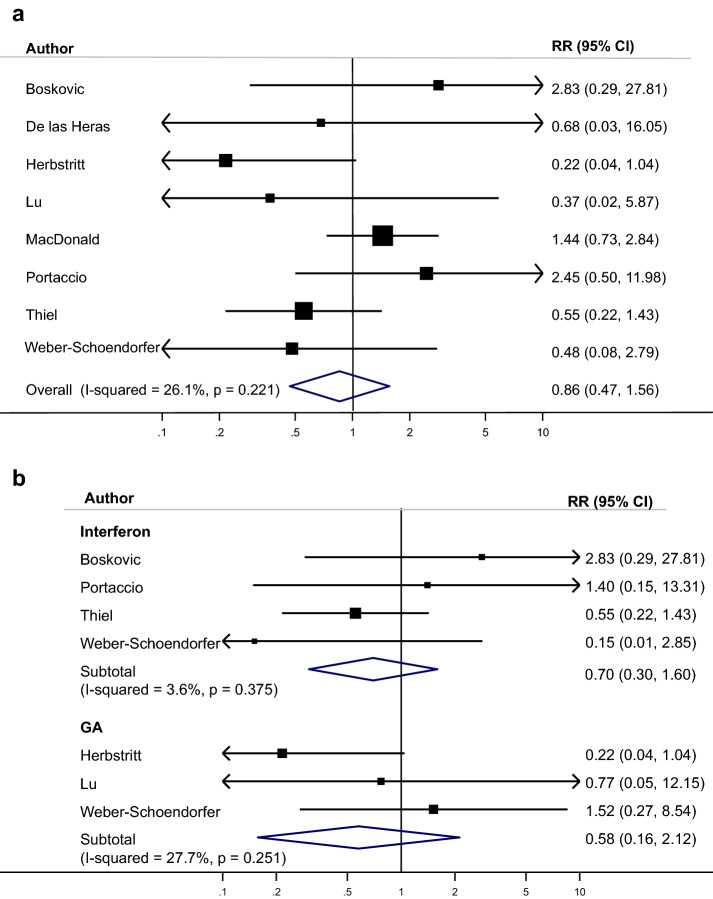
**a** Meta-analysis major congenital malformations: treated MS vs untreated MS. **b** Meta-analysis major congenital malformations: treated MS vs untreated MS stratified by drug

Figures 2, 3 and 4 present meta-analysis for spontaneous abortions (from eight studies), pre-term births (from seven studies) and major congenital malformations (from eight studies) both overall for all MS drugs as well as stratified by drug class. The RR for spontaneous abortion was RR = 1.14, 95% CI 0.99–1.320, for preterm births RR = 0.93, 95% CI 0.72–1.21 and for major congenital malformations RR = 0.86, 95% CI 0.47–1.56 when compared to MS without treatment. The evidence for heterogeneity was low for spontaneous abortions (*I*^2^ = 0%) and major congenital malformations (*I*^2^ = 26.1) and medium for preterm birth (*I*^2^ = 46.0%). There was no evidence of publication bias.

### Pooled prevalence

Table 2 presents the pooled estimated prevalence for spontaneous abortions, premature births and major congenital malformations in untreated as well as treated MS patients. The prevalence for untreated MS patients was: spontaneous abortions 10.9% (95% CI 5.2–18.3) premature birth 12.1% (7.4–17.7) and major congenital malformations 4.2% (2.7–6.1). The prevalence for treated MS patients was: spontaneous abortions 11.6% (7.4–16.7), premature birth 12.12% (9.0–15.6) and major congenital malformations 3.0% (1.8–4.4). The *I*^2^ value was extremely high for each pooled outcome, indicating the extremely wide variation in reported prevalences for these outcomes.

**Table 2:** Meta-analyses of prevalence in the different group studied

	*n*/*N*	Prevalence	LCI 95%	UCI 95%	*I*^2^% Heterogeneity (95% CI)
Spontaneous abortions					
Untreated MS	378/2805	10.9%	5.2%	18.3%	96 (93–97)
All MS Drugs	275/2006	11.6%	7.4%	16.7%	88 (82–92)
Premature birth					
Untreated MS	376/2666	12.1%	7.4%	17.7%	73 (57–84)
All MS drugs	247/2001	12.1%	9.0%	15.6%	92 (86–95)
Major congenital malformations^a^					
Untreated MS	67/1691	4.2%	2.7%	6.1%	61 (21–80)
All MS drugs	33/1040	3.0%	1.8%	4.4%	19 (0–58)

Prevalence takes into account weights. *I*^2^ = 0–40% might not be important, 30–60% moderate heterogeneity, 50–90% substantial heterogeneity, 75–100% considerable heterogeneity

^a^Denominator = number of pregnancies

### Major malformations

The most common major congenital malformations in MS patients exposed to MS drugs were atrial septal defect (ASD) (*N =* 4), polydactyly (*N =* 4) and club foot (*N =* 3). Each case of atrial septal defect was present in patients exposed to different drugs (natalizumab, β-interferon, GA and Mitoxantrone). For the unexposed MS patients most of the studies did not specify what major malformation was present (Table 3). There were only two studies that reported one dysmelia of the tibia and fibula and three ASD [13, 14].

**Table 3:** List of major malformations reported in the exposed patients to MS drugs

*N*	Organs	Number of births with anomaly	Total number of exposed births	Study
6	Cardio-Pulmonar	1 Atrioventricular Canal	25	Weber-Schoendorfer and Schaefer et al. [24]
		1 Atrial septal defect	77	Ebrahimi et al. [16]
		3 Atrial septal defects	226	Thiel et al. [14]
		1 Pulmonary artery stenosis	226	Thiel et al. [14]
4	Renal	1 Ureteropelvic stenosis	226	Thiel et al. [14]
		1 Pyloric stenosis	136	Herbstritt et al. [13]
		2 Ureteral duplication	226	Thiel et al. [14]
			136	Herbstritt et al. [13]
9	Skeletal	4 Polydactyly	75	Portaccio et al. [15]
			226	Thiel et al. [14]
			226	Thiel et al. [14]
			77	Ebrahimi et al. [16]
		1 Macrodactyly	226	Thiel et al. [14]
		1 dysmelia of the tibia and fibula^a^	226	Thiel et al. [14]
		3 Club foot	25	Weber-Schoendorfer and Schaefer et al. [24]
			54	Portaccio et al. [15]
			226	Thiel et al. [14]
2		2 Down syndrome	54	Portaccio et al. [15]
			12	Boskovic et al. [23]
1		1 Hernia**	77	Ebrahimi et al. [16]
1		1 “Abnormality of the X Chromosome”	12	Boskovic et al. [23]
1		1 Wolf Hirschhorn syndrome	226	Thiel et al. [14]

^a^Author does not specify whether this is a major or minor malformation, does not specify what type of hernia

## Discussion

The objective of this meta-analysis was to assess if patients treated for MS had an increased risk of adverse pregnancy outcomes compared to MS untreated patients. The outcomes that were possible to study were spontaneous abortions, pre-term live births and major congenital malformations in live births. The results showed that treatment in general did not appear to be associated with these adverse pregnancy outcomes, with the confidence intervals excluding relative risks greater than 50%. Given the limited number of studies there was not the power to evaluate MS drugs separately in an adequate manner. The results were driven by interferon, GA and natalizumab; therefore, it is not possible to generalize to other drugs such as fingolimod, azathioprine or rituximab. Given the diverse mechanisms by which these DMDs work, understanding each DMD individually is highly important; therefore, there is a need for studies with large sample sizes that present their results stratified by type of drug.

The biggest limitation was the number of available published studies and the small sample sizes in these studies. Small sample sizes might be partially due to labels restricting the use of most MS drugs during pregnancy. There was only one study which used a national commercially insurance population which included a large sample size (574 DMD exposed); however, the authors do not present their data stratified by type of drug, and therefore, it was only possible to stratify the results for interferons, natalizumab and GA. There were 13 studies which we had to exclude, because they had not included controls. This shows that there is a great interest in studying the safety of MS drugs during pregnancy; however, there is a need for studies using internal comparators to assess the risk comparing patients taking MS drugs vs patients not taking medication as well as studying in the same study different MS drugs and taking into account confounding factors.

A limitation of these meta-analyses and pregnancy studies in general is the lack of adjustment for confounding factors such as age, comorbidities, comedications, trimester of exposure and environment. Future studies should consider large databases to conduct observational studies. These databases have the advantage of providing large populations that increase the power and reduce the risk of reporting bias. In addition, by increasing the size of the population, it will be possible to evaluate the risk given by each drug in each trimester of exposure and evaluate other outcomes. It is likely that most of the patients were exposed to DMDs during the first trimester of pregnancy. However, of all the studies identified, there was only one study which focused on the exposure of natalizumab during the first trimester [16]. In addition, there is a need to understand the safety risk when exposed later in pregnancy. For example, hematological abnormalities (anemia and thrombocytopenia), were reported in babies exposed to natalizumab in late pregnancy [17].Concerning the evaluation of other outcomes, Houtchens et al. [18] using an administrative claims database from the United States compared the pregnancy prevalence complications in women with and without MS. They reported that women with MS when compared with women without MS had a higher frequency of premature labor, infections, cardiovascular disease, anemia/acquired coagulation disorders and neurological malformations. They did not study if DMDs increased the risk; however, they estimated that only 20% of the MS patients were exposed to a DMD during pregnancy. Future studies using large databases will be adequate to study the safety related to different outcomes.

Seven out of the ten studies, mentioned the type of major congenital malformations that were present in the patients taking MS drugs; however, only two presented the major congenital malformations in the control groups. In the MS patients exposed to DMDs there was no specific pattern seen. The most common major congenital malformations were atrial septal defect (ASD) (*N =* 4), polydactyly (*N =* 4) and club foot (*N =* 3), which are among the most prevalent birth defects observed in the general population [19]. In addition, the only studies that presented the major congenital malformations in controls also listed ASD. The cases of polydactyly were present in β-interferon and natalizumab patients and the club foot in patients exposed to GA and natalizumab. The biggest study did not list the major malformations [12]. We contacted the authors and they stated that given that the study was performed in claims database it was not permitted to publish non-aggregated data.

It is important to note that not all the studies used the same definition for major congenital malformation. Even for the same type of congenital malformation, some authors considered it to be major and others minor. For example, Portaccio et al. [15] classified hip dysplasia as a major congenital malformation, and Thiel et al. [14] classified it as a minor congenital malformation. In addition, Thiel did not mention if dysmelia of the tibia and fibula was considered major or minor and Herbstritt et al. [13] considered it major. Classifications such as the EUROCAT and The Metropolitan Atlanta Congenital Defects Program (MACDP) need to be considered at all times when performing pregnancy studies [19, 20]. In addition, there is a need for standardization, and especially if secondary sources of data are used, algorithms to define pregnancy outcomes need to be carefully validated.

In addition to DMDs being recommended not to be used during pregnancy, for most DMDs, the European Medicine Agency recommends adding contraception measures and time on contraception after the last dose of treatment (e.g., fingolimod 2 months) [21]. Therefore, evidence of pregnancy occurrence and its outcomes in women with MS exposed to these DMDs might take years to gather through traditional pregnancy registries. In this study, results coming from primary collection in MS pregnancy registries were similar to those from secondary use of data. Secondary use of data sources with broad country coverage have the potential to identify a much higher number of exposed cases in a more timely manner and minimizes loss to follow-up of pregnant women and infants.

A recently initiated IMI2 project named ConcePTION, which the authors are part of, will enable the use of such data [22]. This meta-analysis aims to provide guidance on a future study that will assess the safety of MS drugs during pregnancy. In the future study we will tackle some of the limitations observed in the studies we present in this meta-analysis which include the stratification of different DMDs, standardization of definitions and adjustment for confounding factors. In conclusion, interferons, GA or natalizumab, do not appear to increase the risk for spontaneous abortions, pre-term birth or major congenital malformations. There were very few patients included that were exposed with fingolimod, azathioprine and rituximab; therefore, these results cannot be generalized across drugs. Future studies including internal comparators are needed to enable treating physicians and their patients to decide on the best treatment options.
